# Effects of Manual Therapy and Home Exercise Treatment on Pain, Stress, Sleep, and Life Quality in Patients with Bruxism: A Randomized Clinical Trial

**DOI:** 10.3390/medicina60122007

**Published:** 2024-12-04

**Authors:** Merve Berika Kadıoğlu, Miraç Sezer, Bülent Elbasan

**Affiliations:** 1 Department of Orthodontics, Faculty of Dentistry, Ankara University, Ankara 06560, Turkey; 2 Department of Therapy and Rehabilitation, Physiotherapy Program, Vocational School of Health Services, Bartin University, Bartin 74100, Turkey; msezer@bartin.edu.tr; 3 Department of Physiotherapy and Rehabilitation, Faculty of Health Sciences, Gazi University, Ankara 06490, Turkey; bulentelbasan@gazi.edu.tr

**Keywords:** manual therapy, exercise, bruxism, sleep and life quality, pain, stress, physiotherapy, TMD

## Abstract

*Background and Objectives*: This study aimed to examine the effects of manual therapy (MT) and home exercise (HE) treatments on pain, sleep quality, stress level, and quality of life in patients with bruxism. *Materials and Methods*: The study included 30 bruxism patients ages 18–25 years who were randomly divided into the manual therapy group (MTG) and home exercise group (HEG). Before treatment (T1), all patients were asked to fill out the Pittsburgh Sleep Quality Index (PSQI), Perceived Stress Scale (PSS), Fonseca Anamnestic Index (FAI), and Quality of Life Scale/Short Form-36 (SF-36), and additionally, the number of trigger points (NTP) and pain levels were determined. MT and HE were applied for 8 weeks, and all tests were repeated at the end of treatment (T2). A paired-samples *t* test was used for intra-group comparisons at T1 and T2, and an independent *t* test was used for inter-group comparisons. The statistical significance level was accepted as *p* < 0.05. *Results*: When the results obtained with MT and HE were examined after 8 weeks of treatment, a significant difference was found for all parameters (FAI, PSQI, PSS, SF-36, NTP, activity pain, and night pain levels, *p* < 0.05) except rest pain in HEG. According to the inter-group comparison, a significant difference was found in PSQI, FAI, and NTP (*p* < 0.05). However, it was determined that both groups showed statistically similar effects in terms of pain, perceived stress, and quality of life (*p* > 0.05). *Conclusions*: MT and HE reduce perceived stress and pain intensity and also improve quality of life and sleep in patients with bruxism. HE is as effective as MT in terms of pain, stress quality of life. MT is more effective than HE in improving sleep quality and TMD symptoms. Both manual therapy and home exercise applications can be applied as alternative treatment methods in the treatment of bruxism.

## 1. Introduction

Bruxism is a common parafunctional habit described according to the International Classification of Sleep Disorders as “repetitive jaw muscle activity characterized by clenching or grinding of the teeth and/or supporting or pushing the jawbone” by the American Academy of Sleep Medicine [1]. It can be classified according to the time of onset, etiology, and activity status, and it is often difficult to distinguish between different types of bruxism [2]. Generally, bruxism presents itself in two ways: awake and sleep bruxism. Sleep bruxism is a masticatory muscle activity characterized as rhythmic (phasic) or non-rhythmic (tonic) during sleep, while awake bruxism is a masticatory muscle activity characterized by repetitive or sustained tooth contact and/or by bracing or thrusting of the mandible during wakefulness [3]. According to a systematic review of the literature, bruxism is a common condition, with estimated prevalence rates for the general adult population of 8–32% for awake bruxism and 12.8–22.2% for sleep bruxism [4,5,6]. It has been reported that the prevalence of bruxism decreases with age: 14–20% in children, 13% in young adults between the ages of 18 and 29, and 3% in adults over the age of 60 [4,7,8]. Although the periods of teeth grinding are short intervals, it may cause serious problems such as pain in the head, temporomandibular joint (TMJ), hypertrophy in the masticatory muscles, temporomandibular joint disorders (TMDs), tooth wear, sensitivity and pain, fracture, or failure of dental implants [9,10].

Although the etiology is not known exactly, there is a consensus that it is multifactorial. Emotional stress is one of the important factors leading to bruxism. In a study, it was shown that a stressful life has an important effect in explaining clenching during the day [11]. In addition to stress, there are studies showing that psychiatric disorders, especially depression and anxiety disorders, accompany TMD and bruxism [12]. Studies have shown that insomnia symptoms increase 1.7-fold and stress-induced sleep time shortens in chronic moderate or severe stress conditions, and it has been stated that high levels of stress cause an increase in depressive symptoms and muscle weakness and a shortening in total sleep time [13,14,15]. Therefore, it is thought that disturbances in sleep quality may cause disorders related to the masticatory system both directly and indirectly. In this context, studies have found that sleep problems or psychological disorders are present simultaneously in most patients with bruxism [16,17]. For these reasons, evaluation of both sleep quality and stress factors in the assessment of bruxism is important in terms of accurate diagnosis and optimal treatment planning.

There is currently no effective treatment that completely “cures” or “stops” bruxism permanently [2,18]. The current approach in the treatment of bruxism is aimed at controlling symptoms, preventing complications that may occur, preserving teeth or existing restorations, reducing bruxism activity, and relieving pain. For this, it is emphasized that patients should be evaluated and treated by a multidisciplinary team consisting of a dentist, physiotherapist, psychologist, and psychiatrist [19]. In this context, pharmacological agents such as local botulinum toxin A injections; neurological/psychiatric drugs (such as L-dopa and clonazepam); psychological therapies such as biofeedback, hypnotherapy, cognitive therapy, behavioral therapy, and stress and relaxation management; various dental strategies such as stabilization splints and occlusal adjustments; and a wide range of physiotherapy applications have been used to manage bruxism [16,20,21,22,23,24,25,26]. Among these techniques, the use of occlusal appliances is one of the most well-established treatments to manage the consequences of sleep bruxism in a reversible and non-invasive manner, but still its effect mechanism is not fully understood, and studies have reached controversial results regarding its influence on bruxism [18,27,28,29]. In their recent meta-analysis study, Ferreira et al. found that occlusal splint treatment did not influence muscle activity, muscle volume, bite force, or masticatory performance in bruxism patients [27]. In this context, physical therapy applications aiming to directly affect muscles and ligaments gain great importance in the treatment of bruxism, which is defined as “repetitive muscle contractions”. Electrotherapy agents, exercise therapy, acupuncture, trigger point stimulation therapy, dry needling, and manual therapy applications have taken their place in the literature among the methods applied in the field of physical therapy [16,20,24,30,31,32,33,34,35]. Although the quality and number of studies on the effectiveness of these methods are limited, it has been reported that they generally reduce pain in the muscles and increase mouth opening [20]. Exercise therapy, one of the physical therapy applications, has an important place in the rehabilitation of musculoskeletal disorders. This method aims to decrease inflammation, increase coordination of muscle activity, promote tissue repair and regeneration, relieve pain, and restore normal function [36]. In addition to the special exercises known as Rocabado exercises, which are used in the treatment of temporomandibular joint disorders, various studies have been performed in which mobilization, coordination, posture, and relaxation exercises were used for the treatment of disorders related to the masticatory system, and it has been emphasized that studies on this subject should be continued [31,34,37].

Manual therapy is another form of physiotherapy that is performed with the hands, unlike exercise therapy. With this non-invasive method, muscles, joints, ligaments, fascial tissues, and nerves are treated with special maneuvers and techniques. Some of these techniques include joint and soft tissue mobilizations, myofascial releases, trigger point release techniques, massage techniques, and intramuscular stretches. Soft tissue mobilizations, muscle strengthening and stretching exercises, and posture exercises within the scope of manual therapy and exercise therapy applied in the treatment of patients with bruxism aim to reduce muscle tone and activity, provide reflexive relaxation by stretching shortened muscles, increase local blood circulation and metabolic activity, and thus reduce pain [18,38]. Thus, manual therapy is expected to alleviate muscle symptoms such as pain, tension, stiffness, and fatigue; increase mandibular range of motion; and improve jaw function [39]. In this context, studies show that manual therapy can be applied clinically to increase the range of motion in TMJ [16] and to decrease pain severity [34].

In studies, manual therapy and exercise therapy were applied alone or in combination with different methods in patients with bruxism or TMD, and it was stated that they were effective in reducing pain and increasing mouth opening and TMJ functions [16,20,34,35,37,39,40]. However, most studies have only examined the effect of manual therapy and/or exercise treatments on pain, mouth opening, and jaw function. Psychological changes with the treatment of bruxism such as stress, sleep quality, and quality of life, which have a serious cause and effect relationship with each other, have been examined in limited studies [30,35,41,42,43]. In addition, instead of applying only manual therapy or exercise therapy alone, many methods such as cognitive therapy, occlusal splint therapy, etc. were applied together in these studies. This made it difficult to understand from which treatment method the results were obtained. No study has been found that separately examines how manual therapy or exercise therapy applications for the treatment of bruxism lead to changes in pain, sleep quality, stress level, and quality of life, although they are closely related to each other.

In this context, the aim of this study is to comparatively examine the short-term effect of manual therapy and home exercise treatments on pain, sleep quality, stress level, and quality of life in patients characterized with bruxism.

## 2. Materials and Methods

### 2.1. Ethical Approval, Informed Consent, and Clinical Trial Registration

The institutional ethical committee of the Faculty of Social and Human Sciences, Bartın University, approved the present study (approval number: 2024-SBB-0289, decision date: 22 April 2024). All the study procedures were performed in accordance with the Declaration of Helsinki [44]. The patients included were asked to participate in the study protocol, and written informed consent indicating that they were willing to participate in the study was obtained. The study design of this trial was registered on ClinicalTrials.gov (NCT06610435).

### 2.2. Sample Size Calculation

The G*Power (3.1.9.7) program was used to determine the number of participants to be included in the manual therapy and exercise groups of the study. The sample size calculation was based on a power analysis. The sample size was established based on a similar previous study comparing the effects of different treatment methods on masticatory muscle activity, pain, posture, psychological status, and quality of life in temporomandibular joint disorder which include 14 participants in each group [43]. Based on this previous study, the sample size for each group was calculated as at least six people, with a power of 0.80, a confidence interval of 95%, and α = 0.05 (Type-I error) (see Supplementary S1: Power analysis for sample size calculation). Since two main treatment groups were planned for the study protocol, a total of 12 people were required to participate in the study. However, considering the possible losses that may occur, 35 people were included in the study.

### 2.3. Trial Design, Allocation, and Randomization

**Figure 1:**
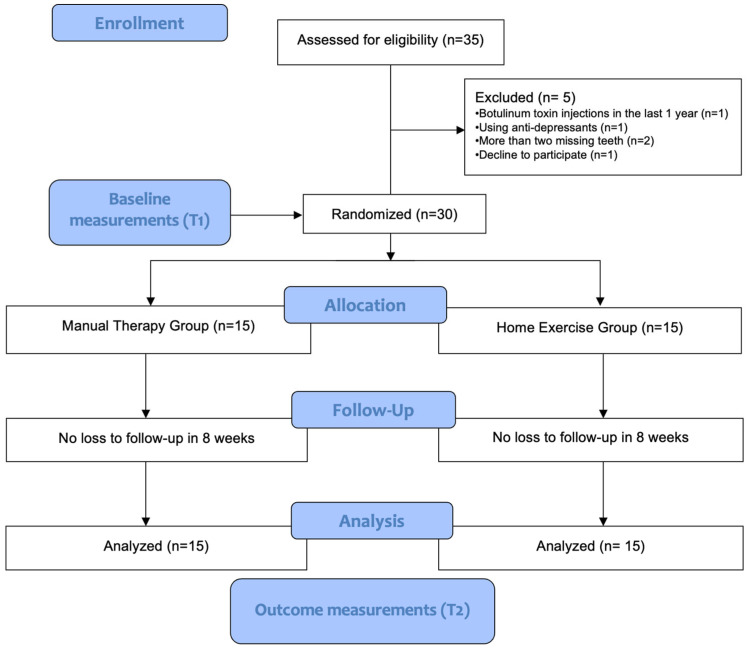
Study design and CONSORT flow diagram.

The current single-center study was a single-arm, parallel-group, randomized clinical trial with blinded assessment. The study protocol was carried out in accordance with the CONSORT (2010 checklist of information to include when stating a randomized trial, Consolidation Standards for Reporting Studies) statement [45]. The study was planned in such a way that patients included in the study protocol were equally assigned to one of two different main treatment groups: the manual therapy group (MTG) and home exercise group (HEG). The study included volunteer students studying at Bartın University School of Health Services between the ages of 18 and 25 who answered “yes” to at least two questions on the bruxism questionnaire (Table 1) [46,47]. Individuals who were pre-diagnosed with “bruxism” in the preliminary evaluation of the questionnaire were clinically evaluated by an expert orthodontist in terms of clinical signs of bruxism: abnormal tooth wear on the occlusal surfaces of the teeth, abfraction, gingival recession and/or cervical defect, tongue indentations, damage to the inside of the cheek, tense facial and jaw muscles, muscle sensitivity, and masseteric hypertrophy upon bidigital palpation [1,3,48]. Individuals who had at least two of these clinical findings in addition to the bruxism questionnaire results were included in the study. Subsequently, participants were randomly assigned to either the manual therapy group (MTG) or home exercise group (HEG) (Figure 1). In this study, randomization was performed using a simple randomization method, and the randomization process was carried out by online randomization software (https://www.randomizer.org; accessed on 15 July 2024) [49]. The study procedures were conducted from May 2024 to July 2024 at a single center (Bartın University, School of Health Services, Bartın, Türkiye). Evaluating the participation and eligibility criteria of the patients was performed by an experienced orthodontist and physiotherapist. In addition, in order to ensure blinding, baseline and final outcome measurements were performed by researchers other than the physiotherapist performing manual therapy.

### 2.4. Patient Selection and Eligibility Criteria

Thirty-five patients who volunteered for the study and were confirmed to have “bruxism” by the bruxism questionnaire and clinical evaluation were re-evaluated for exclusion criteria. The exclusion criteria for participants were as follows:

- Being characterized by a neurological disease,
- Botulinum toxin injection into the masticatory muscles within the last year,
- The use of antidepressants that affect the central nervous system,
- The application of occlusal splint therapy,
- More than two molar teeth missing in the posterior side.

Thirty individuals who met the inclusion criteria were randomly selected via online randomization software (https://www.randomizer.org; accessed on 15 July 2024) and divided into two equal groups: the manual therapy group (MTG) (*n* = 15) to receive manual therapy and the home exercise group (HEG) (*n* = 15) to receive exercise therapy. All individuals included in the study received their planned treatments, and a final evaluation was made on all individuals without any loss at the end of the 8-week period. The patient selection procedure and the CONSORT flow diagram are presented in Figure 1.

### 2.5. Baseline and Outcome Measures and Blinding

After obtaining demographic information from all patients, they were asked to fill out the Pittsburgh Sleep Quality Index (PSQI), Perceived Stress Scale, Fonseca Anamnestic Index (FAI), and Short Form-36 (SF-36) Quality of Life Scale, and the number of trigger points and pain levels were determined at baseline (before treatment—T1). The programmed treatments were applied for 8 weeks in both groups, and at the end of 8 weeks, all tests were repeated and re-evaluated (after treatment—T2). In order to ensure blinding, the baseline and final assessments were performed by other investigators, instead of the investigator who applied manual therapy (M.S.), without knowing which group the participants were included in.

- Bruxism questionnaire: According to the survey questions prepared with reference to the studies of Pintado et al. and Shetty et al., it was stated that individuals who answer “Yes” to at least two of the questions in the questionnaire were characterized by bruxism and can be called “bruxist” [46,47] (Table 1).
- Fonseca Anamnestic Index (FAI): It was developed in 1994 by Fonseca et al. and consists of 10 questions investigating pain in the head and TMJ (Table 2). Turkish validity and reliability of the test was performed by Kaynak et al. [50]. The questionnaire includes various questions about joint, head, and neck pain; joint movements; parafunctional habits; impaired occlusion; and emotional stress. Participants were asked to answer “Yes” (10 points), “Sometimes” (5 points), or “No” (0 points) to each question, and TMD was classified as none, mild, moderate, or severe according to the total score [50,51,52].
- Pittsburgh Sleep Quality Index (PSQI): It is a 24-question scale developed by Buysse et al. in 1989 that evaluates sleep quality in the last month [53]. A Turkish validity and reliability study was performed in 1996 [54], and the last five questions are used only for clinical evaluation. The sum of the scores of seven components, subjective sleep quality, sleep latency, sleep duration, habitual sleep efficiency, sleep disturbance, sleep medication use, and daytime dysfunction, gives the total score index. The total PSQI score ranges from 0 to 21; a score above 5 points indicates poor sleep quality; a score of 5 points or less indicates good sleep quality. The PSQI is one of the most commonly used scales for evaluating sleep quality. The validity of the use of this index in sleep disorders has been tested before, and it has been frequently used in studies on bruxism [20,55,56,57,58].
- Perceived Stress Scale: It was developed by Cohen, Kamarck, and Mermelstein in 1983 and consists of 14 items. Its Turkish validity and reliability was performed by Eskin et al. [59], and this scale has been used in many studies on bruxism [60,61,62,63,64]. The scale consists of two sub-dimensions: stress/discomfort perception and self-efficacy perception. Participants rate the stress they perceive on the scale as “0” never, “1” almost never, “2” sometimes, “3” often, and “4” very often. The stress level perceived by individuals is determined by summing the scores obtained from the items. A score between 0–56 points is obtained from the scale, and the higher the score, the higher the perceived stress level [59].
- Quality of Life Scale/Short Form-36 (SF-36): SF-36 was developed by the Rand Corporation to obtain information about the health status of the individual. It was translated into Turkish by Koçyiğit and his colleagues, who conducted a validity and reliability study [65]. It consists of eight sub-dimensions and 36 items. The sub-dimensions consist of physical function, social function, physical role difficulty, emotional state difficulty, mental health, energy/vitality, pain, and general perception of health. A score of “0” represents the worst health status, while “100” represents the best health status. Each sub-dimension is evaluated individually without calculating the total score, and in our study, general health perception was evaluated.
- Trigger Point Evaluation: While evaluating the trigger points in the masticatory and neck muscles, the tense muscle was palpated with a fingertip [66]. Palpation was performed along the long axis of the tense muscle, and the most sensitive point was determined. Sudden reaction or vocal response of the patient with light pressure applied to this point and the presence of reflected pain in a region distant from this region indicated the presence of a trigger point. Evaluation was performed in 14 muscles, and the muscles with trigger points and the total number of trigger points were recorded [66].
- Pain level—Visual Analog Scale: A visual analog scale (VAS) was used to assess the severity of pain related to bruxism. A VAS line was drawn on a 10 cm long horizontal line (0 = no pain and 10 = most intense pain), and the patient was asked to mark the pain intensity they perceived at rest, active use, and at night on this VAS line. The pain intensity of the individual was recorded by measuring the distance marked on the line in millimeters. It has been reported that the Turkish version of the VAS is a valid and reliable measurement tool for the evaluation of musculoskeletal disorders [67].

### 2.6. Interventions

In both treatment groups, various stretching, relaxation, and strengthening techniques were applied to achieve similar effects on similar muscle and connective tissue groups using different techniques. Since it usually takes 6–8 weeks for the targeted muscle and connective tissues to adapt and heal, increase blood flow, and improve tissue elasticity, we planned to apply manual therapy and home exercises for 8 weeks in the study [68,69].

A. Manual therapy group (MTG): The term manual therapy includes a wide variety of detailed applications ranging from joint-oriented applications (joint mobilizations and/or manipulations) to soft tissue techniques (muscle stretching or trigger point therapy) to therapeutic exercises [32]. Manual therapy in our study was used to restore normal temporomandibular joint range of motion, reduce local ischemia, stimulate proprioception, break fibrous adhesions, stimulate synovial fluid production, and reduce pain [16]. In this context, various soft tissue and joint mobilizations, intramuscular stretches, trigger point treatments, and intraoral applications for the temporomandibular joint were performed in 15 randomly selected bruxism patients.
  First, mild distraction to the vertebrae was applied with the “bridging” technique, and also the activation of the parasympathetic system was applied by stretching the suboccipital and fascialized neck muscles. After myofascial release techniques for the neck fascia, passive stretching of the trapezius and levator scapula muscles, ischemic compression, and friction applications for trigger points and fibrositis formations were performed. The scalene and sternocleidomastoid (SCM) muscles were palpated, and when trigger points were detected, compression was applied to these points for 90 s, and the muscles were allowed to relax. Intramuscular stretching techniques were applied to reduce muscle tone and resolve spasm; the same procedure was performed for the infrahyoid and suprahyoid muscles. After mobilization of the hyoid bone, stretching was applied especially to the posterior part of the digastric muscle.
  A post-isometric relaxation technique was applied to the jaw muscles, aiming to relax the muscles with increased tone through autogenic inhibition. Individuals were asked to open their mouths to the end point of the joint movement and perform isometric contractions in the opposite direction of the movement [66,70]. After this application, the masticatory muscles were localized and treated. Soft tissue mobilization techniques, myofascial release, intramuscular stretching, ischemic compression of trigger points, and friction massage were applied to the medial pterygoid, masseter, and anterior temporalis muscles from the lower region of the angulus mandible. After these procedures, intraoral applications were started; medial pterygoid, lateral pterygoid, masseter muscles, and the tendon of the anterior temporalis muscle were mobilized. Patients were informed before these applications, which were painful at first, and it was observed that their tolerance to pain increased as the tissues relaxed. The manual therapy procedure, which lasted approximately 40 min and was performed in the supine position, was performed twice a week for 8 weeks by the same specialist physiotherapist.
B. Home exercise group (HEG): The exercise group consisted of 15 randomly selected bruxist individuals and aimed to reduce pain; decrease involuntary contractions of the masticatory muscles; increase their nutrition, flexibility, and coordination; and strengthen weak muscles with exercise therapy.
  Opening and closing the mouth by placing the tongue on the upper palate; stretching exercises for masseter, temporalis anterior, and neck muscles to relax the jaw and facial muscles; isometric exercises and the self-post isometric relaxation technique; posterior tilt; and posture exercises were given to strengthen the jaw and neck muscles [34,37].
  All individuals in this group were taught the programmed exercises practically by a specialist physiotherapist on the first day of the evaluation and were asked to repeat the home exercises three times a week for 8 weeks. They were asked to perform the exercises while sitting in a chair with back support, maintaining their position in front of a mirror, and monitoring themselves and their movement symmetry. Each movement comprising the exercises was performed for 10 repetitions and completed in approximately 25 min.
  In addition, a video explaining the exercises in detail was shared with all participants in the group to ensure that the exercises were not forgotten and to ensure accuracy and standardization among the participants. Individuals were called by phone every week to check whether they complied with the exercise frequency and performed the exercises regularly.

### 2.7. Statistical Analysis

Bruxist patients who underwent manual therapy and home exercise were evaluated before and after the intervention, and the statistical analysis of the data obtained was performed in IBM SPSS Statistics V26 software (Statistical Package for the Social Sciences version 26 software, IBM Corporation, Armonk, NY, USA).

Descriptive analyses, “Mean ± Standard Deviation (X ± SS)” and percentage (%) values were calculated for numerical data. Differences between categorical variables were analyzed by Pearson’s Chi-squared test.

The normal distribution of the groups was tested with the Shapiro–Wilk test, and it was determined that the data were normally distributed (*p* > 0.05). Detailed information about the Shapiro–Wilk analysis results and normality distribution value table are given in Supplementary S2.

Since normality was ensured, parametric tests were used for inter-group and intra-groups comparisons. A paired samples *t* test was used for intra-group comparisons before (T1) and after (T2) intervention, and an independent *t* test was used for inter-group comparisons. For all analyses, 95% confidence intervals were presented for each measurement time point. A *p*-value of <0.05 was considered statistically significant for all analyzed data.

## 3. Results

Thirty-five patients were evaluated for eligibility. Five patients were removed from the study due to meeting the exclusion criteria, and the remaining 30 patients were randomly assigned to one of two groups (Figure 1). At the end of 8 weeks, treatment was completed without any loss in the groups, and final evaluations were made.

Sociodemographic characteristics of the individuals in the manual therapy and home exercise groups are presented in Table 3. It was determined that there was no statistically significant difference between the two groups in terms of gender, age, eating habits, headache, neck stiffness, or parafunctional habits (*p* > 0.05), and the groups were homogeneously distributed. In addition, it was determined that the majority of the participants in both groups were female (MTG: 73.3%; HEG: 66.7%) and suffered from headache (86.7% both), and all of them had parafunctional habits (such as gum chewing, lip biting, nail biting, and the like).

The findings related to sleep quality, quality of life, and perceived stress level of the patients in the manual therapy and exercise groups are given in Table 4. When the changes occurring with treatment in both the manual therapy and home exercise groups were compared separately (T1–T2), it was seen that sleep quality (*p* < 0.001 and *p* = 0.016, respectively), quality of life (*p* < 0.001 and *p* = 0,01, respectively), and perceived stress (*p* = 0.017 and *p* = 0.003, respectively) scores changed statistically significantly with treatment in both groups. Accordingly, it was determined that sleep quality increased, quality of life improved, and stress level decreased with treatment in both groups. When the comparison between groups was examined, it was observed that there was no statistically significant difference between the manual therapy and home exercise groups in terms of sleep quality (*p* = 0.763), quality of life (*p* = 0.302), or perceived stress level (*p* = 0.207) before treatment (T1). After the treatment (T2), while there was no difference between the groups in terms of quality of life (*p* = 0.192) and perceived stress (*p* = 0.875), there was a statistically significant difference between the groups in favor of the manual therapy group only in terms of sleep quality (*p* = 0.005).

The findings related to the Fonseca Anamnestic Index (FAI), bruxism questionnaire, number of trigger points, and pain of the patients in the manual therapy and exercise groups are given in Table 5. According to these results, statistically significant changes were obtained for all data both with manual therapy and exercise therapy (except rest pain in the exercise group). In both groups, FAI, bruxism questionnaire score, number of trigger points, resting pain (except exercise group), activity pain, and night pain decreased statistically significantly (*p* < 0.05). In the home exercise group, the decrease in rest pain with treatment was found to be statistically nonsignificant. When the comparison between the groups was analyzed, it was determined that there was no statistically significant difference between the manual therapy and home exercise groups (except bruxism score) before treatment (T1), and the groups were statistically similar (*p* > 0.05). There was a statistical difference between the groups due to the fact that the bruxism score was higher in the manual therapy group (MTG: 4.66 ± 1.17; HEG: 3.60 ± 1.35; *p* = 0.029) at baseline (T1). After treatment (T2), it was determined that there was no statistically significant difference between the groups in terms of the bruxism questionnaire (*p* = 0.100), resting pain (*p* = 0.315), activity pain (*p* = 0.335), or night pain (*p* = 0.125), and there was a statistically significant difference between the groups in favor of the manual therapy group only in terms of FAI (*p* = 0.047) and number of trigger points (*p* = 0.016).

## 4. Discussion

In this study, we aimed to examine the short-term effects of manual therapy and home exercise therapy on pain, perceived stress, sleep, and quality of life in patients characterized by bruxism. Accordingly, one group of randomly selected bruxist patients received manual therapy (*n* = 15) by a physiotherapist, while the other similar group received a self-administered home exercise program (*n* = 15) for a period of 8 weeks, and pre-treatment and post-treatment evaluations were performed. In both groups, which showed similar values except for the bruxism questionnaire at the beginning, statistically positive changes were obtained in terms of all parameters with the treatment. In this respect, after 8 weeks of treatment, perceived stress, number of trigger points, rest pain (except for the home exercise group), activity pain, and night pain decreased in both the manual therapy and exercise groups, while significant improvements were noted in sleep quality, quality of life, the bruxism questionnaire, and Fonseca Anamnestic Index scores.

A wide range of treatment modalities have been defined for the treatment of bruxism, which has many etiological factors, including drug therapy, physical therapy, manual therapy, dry needling, occlusal splinting, botulinum toxin applications, and biobehavioral therapy [16,22,23,24]. However, the most effective treatment method is still unclear.

Manual therapy and home exercises, which have many advantages such as being easily accessible, non-invasive, and economical, have recently been frequently used in the treatment of bruxism and TMD, and their effects on many symptoms such as pain, mouth opening, quality of life, and sleep have been examined in different ways [16,22,30,34,71,72]. In our study, it was observed that sleep and quality of life significantly increased, while bruxism level, pain, perceived stress, and TMD symptoms decreased as a result of 8-week manual therapy and home exercise treatments in patients with bruxism.

When the sociodemographic data at baseline were analyzed, it was found that the groups showed statistically similar characteristics, which is very important in terms of ensuring the homogeneity of the groups. Additionally, it is an important finding that the majority of the individuals participating in the study (86.6%) complained of headache, and all of them exhibited additional parafunctional habits. Carra stated that individuals with sleep bruxism reported three times more headache attacks than those without bruxism and that there may be a potential link between inadequate sleep and headache occurrence [73].The relationship between bruxism and headache has been extensively discussed, and a consensus has been reached that there is a significant relationship between tension-type headaches, craniofacial pain, migraine, and sleep bruxism [63,74]. Although the cause–effect relationship has not yet been fully determined, it is thought that the pain may resemble post-exercise muscle pain caused by mechanical overload, and inflammation caused by muscle hyperactivity is effective in pain occurrence [75]. In addition, the fact that repetitive activities related to parafunctional habits are thought to be an important factor that maintains pain supports our finding that all individuals participating in our study were characterized by headache and parafunctional habits [76].

In our study, the complaint of headache, which was seen in almost all patients at the beginning of treatment, was reduced or completely eliminated with both manual therapy and home exercise. Moreover, there was a significant decrease not only in rest, activity, and night pain, but also in the number of trigger points. Similar to our study, Mihaiu et al. reported that psychological counseling in addition to manual therapy and home exercise provided significant improvement in terms of pain, anxiety, perceived stress, and bruxism in individuals with headache and emphasized that a multidisciplinary approach is more effective [63]. In other studies with a long-term follow-up, it was reported that manual therapy targeting the orofacial region in addition to the cervical region was more effective in reducing pain in patients characterized by headache and TMD and showed long-term positive results [34,72]. La Touche et al. reported that cervical spine mobilization reduced pain intensity, increased pain thresholds, had a stimulating effect on the sympathetic system, and reduced stress in TMD patients with myofascial-induced cervico-craniofacial pain [77]. In this respect, our study supports the literature.

In another study on manual therapy, it was found that a soft occlusal repositioning splint was ineffective in terms of pain and mouth opening in TMD patients characterized by disc displacement with reduction, while the application of active exercises in addition to manual therapy was reported to be significantly effective in reducing pain and increasing mouth opening [78]. Nicolakis et al. reported a 75% reduction in pain level in TMD patients with non-reducing disc displacement by applying only home exercises, including active and passive jaw movement exercises, correction of body posture, and relaxation techniques [79]. In a meta-analysis study, Zhang et al. reported that occlusal splinting and exercise therapy were similarly effective for pain relief and emphasized the need for more randomized clinical studies [26].

In another study in which exercise therapy and manual therapy were compared, posture exercises were applied to one group of patients with myofascial TMD, while temporomandibular joint and soft tissue mobilizations were applied to the other group in addition to the exercise program, and as a result, a decrease in pain intensity and improvement in head posture were recorded in both groups after treatment [34]. It has been reported that the combined application of manual therapy and exercises is more effective in reducing pain than exercise alone [34]. In the study in which many applications such as orofacial manual therapy, motor control training, exercise, and Bruxism neuroscience education were performed at the same time, a decrease in pain and improvement in functions were found, but it was not fully understood which method was most effective [80].

In our study, the effects of manual therapy and home exercise treatment on pain were compared between the groups. Although numerically more improvement was achieved with manual therapy in general, only the decrease in the number of trigger points was found to be statistically significant in favor of manual therapy between the groups. To the best of our knowledge, we have not encountered a study that directly compares the effect of manual therapy and home exercises solely. One short-term study found that manual therapy in conjunction with home physical therapy is more effective than home physical therapy alone for the treatment of TMD, particularly with regard to decreasing pain and increasing pain-free maximum mouth opening [81]. It is thought that this difference between the studies is due to the synergistic effect of using manual therapy and home exercises together. According to the results obtained in our study, it can be concluded that home exercises are as effective as manual therapy in reducing pain in bruxism treatment.

As mentioned before, bruxism is affected by many factors, with stress and sleep quality among the leading factors. According to a 2009 review, it was stated that there was some relationship between bruxism and anxiety, stress susceptibility, depression, and other personal characteristics [82]. The perceived stress level in patients with bruxism was found to be higher than those without bruxism [83]. Emotional distress, stress, anxiety, and depression have been associated with the occurrence of signs and symptoms of TMD in various populations, and these symptoms were observed more frequently in patients with myogenic TMD [84]. It has been reported that especially stress and anxiety may lead to microtraumas in the TMJ and changes in muscle tissue by triggering parafunctional habits as well as increased muscle tone [85]. In a study conducted in our country, a positive and strong correlation was found between stress, anxiety, and depression levels in patients with TMD [86]. In studies conducted on bruxist patients with and without orofacial pain, moderate/severe depression and non-specific physical symptoms were shown to be significantly more common and it was thought that pain may trigger depression [17,87].

In addition, in a study on TMD, it was reported that manual therapy applications applied to the temporomandibular and cervical region caused a decrease in depression and anxiety levels [88]. Also, it was stated that depression and anxiety in patients with TMD may be caused by other symptoms such as pain rather than stress. In a study in which primary headache, bruxism, and psychological status was examined, it was found that manual therapy applications were effective at reducing stress and perceived pain, and it was reported that the application of counseling in addition to manual therapy created even more positive results [63]. Tuncer et al. found that the application of manual therapy with home exercises was more effective in terms of improvement in posture, pain, and stress than the application of home exercises alone [34]. In support of previous studies, the results of this study showed that both treatment modalities similarly led to a statistically significant reduction in the level of perceived stress in patients with bruxism.

High levels of stress are associated with sleep disturbances, reduced nighttime sleep duration, high levels of fatigue, and depressive symptoms. High levels of perceived stress and sleep disturbances are important predictors of depressive and physical symptoms. It has been reported that daytime sleepiness and depressive and physical symptoms are more common in people with poor sleep quality compared to good sleepers [15]. Serra Negra et al. reported that poor sleep quality was a very important factor in dental students with bruxism, and Neu et al. reported that sleep efficiency of patients with sleep bruxism decreased significantly, sleep interruptions increased, and sleep quality was worse [89,90]. In a study investigating the effects of different physiotherapy applications on sleep quality, TENS, massage, and trigger point treatments were applied to patients with bruxism, and it was observed that the PSQI score of patients who received massage and trigger point treatment decreased and sleep quality increased after 6 weeks [30]. In our study, it was observed that the PSQI values and perceived stress levels decreased significantly with both manual therapy and home exercise, supporting the literature. In addition, when a comparison was made between groups, manual therapy was found to be statistically significantly more effective in increasing sleep quality, while manual therapy and home exercises were found to be similar in terms of perceived stress. In this context, the results we obtained that manual therapy and home exercise methods used in the treatment of bruxism positively affect stress and sleep quality support the literature.

Quality of life has been defined as a concept that affects the level of satisfaction of the person in daily life and reflects the response to diseases and the physical, mental, and social effects of life [91]. In this regard, sleep disorders, stress, anxiety, chronic pain, physical disorders, and dysfunctions negatively affect quality of life and may lead to the development of bruxism. It has been reported that sleep quality is worse, oral health-related quality of life is lower, and anxiety and depression levels are higher in patients with bruxism [57,91]. In studies, it has been stated that chronic pain, which is the most common symptom of TMD, may cause various psychological disorders such as depression, stress, and anxiety, which may decrease the quality of life of patients, and therefore, patients with TMD have a lower quality of life [92,93]. In a clinical study conducted by De Resende et al. to compare the effects of conservative treatments on pain, quality of life, and sleep quality in patients with TMD, it was reported that occlusal splint treatment, manual therapy, and patient education resulted in significant improvements in pain, quality of life, and sleep quality [41]. In a study conducted in patients with muscle-induced TMD, it was stated that patient education and exercise therapy were effective in reducing pain and improving quality of life in both the short and medium term [94]. Izzetti et al. applied occlusal splint therapy and manual therapy together in patients with TMD who showed high anxiety and depression at the beginning of treatment and reported that the initial low level of quality of life increased with treatment, and anxiety, pain, and depression decreased [42]. In another study conducted in individuals with bruxism, it was observed that manual therapy and kinesio-taping in addition to manual therapy contributed to sleep quality and quality of life at a similar rate, while the combined application of kinesio-taping and manual therapy was more effective at reducing pain [19]. In the study in which quality of life was evaluated with SF-36, it was concluded that TENS, exercise, and manual therapy applications among physical therapy methods were significantly effective in improving the quality of life of patients [43].

According to our study results, the lower quality of life of the patients with bruxism at baseline (MG: 45.00 ± 11.33; EG: 51.33 ± 20.39) supports the literature. In addition, in both groups, a significant improvement was observed after the treatments in terms of quality of life (MG: 67.33 ± 21.11; EG: 58.00 ± 16.88), which was below the standards of Turkey (norm values; Men: 73.6 ± 14.9, Women: 69.1 ± 16.9) before treatment [95]. However, the quality of life after treatment was still below the standards of Turkey. Furthermore, according to the intergroup comparison, it was determined that the contribution of manual therapy and home exercise to the quality of life of bruxist individuals was statistically similar, although numerically more improvement was observed with manual therapy.

To determine the presence and severity of TMD symptoms, the Fonseca Anamnestic Index (FAI) is considered a reliable tool for screening in population studies [50]. In the literature, the rate of TMD has been reported between 42% and 73% in studies using the FAI [96,97]. In a recent study conducted on 2580 volunteers in our country, it was reported that 70% of the participants were characterized with mild-to-severe TMD according to FAI and that the presence of bruxism, parafunctional habits, anxiety, and depression were important risk factors for TMD symptoms [98]. Erdil et al. reported that FAI decreased from 50.00 ± 19.58 to 42.00 ± 20.72 at the end of 6 months with Botulinum Toxin A injection for the treatment of bruxism [21]. In our study, while there was severe TMD according to FAI at the beginning of the treatment (MTG: 67.67 ± 11.32; HEG: 71.33 ± 13.02), it was observed that both groups regressed to mild TMD (MTG: 34.33 ± 13.61; HEG: 44.33 ± 12.80) at the end of the treatment. In addition, according to the comparison between the groups, the improvement in the manual therapy group was found to be statistically significantly higher. When compared with our study results, it can be said that both manual therapy and exercise therapy provided more improvement in TMD symptoms compared to Botox treatment [21]. In addition, it is thought that improvement in FAI is closely related to improvement in pain, stress, quality of life, and sleep quality.

In general, although there are studies examining the various effects of manual therapy and exercise therapy, they either compared the effectiveness of manual therapy and exercise therapy in combination with different treatments instead of applying them alone, or only limited parameters such as pain or mouth opening were examined [30,35,42,63,81]. In our study, manual therapy and exercise therapy were programmed to affect similar muscle groups for similar durations (75–80 min per week) and were applied completely independently of each other in order to eliminate the synergistic effects that may arise. Thereby, we aimed to make a more precise distinction between which treatment was effective at which level. Undoubtedly, the inclusion of patients with bruxism who did not receive any treatment as a control group could have increased the accuracy of the results obtained. However, we thought that it would not be in accordance with ethical rules to keep individuals who already have pain, sleep disturbances, stress/anxiety, and low quality of life in addition to the complaint of bruxism for a long period of 2 months without any treatment. We also felt that the inclusion of individuals without bruxism as a control group would give the wrong direction to the results. For this reason, two active treatment groups were included in the study, but a single treatment protocol was applied without any combined treatment in order to preserve the purity of the results.

In addition, not only the effect on pain but also the effect on different parameters such as stress, sleep, and quality of life, which are closely related to each other, was examined. Although these evaluated parameters are subjective data, we tried to increase the accuracy of the results by using scientifically accepted questionnaires such as PSQI, Fonseca Anamnestic Index, Perceived Stress Scale, and SF-36, the reliability and validity of which have been tested already [30,43,50,54,59,65,98]. In addition, there has been no validation study on the use of these indexes and questionnaires in patients with bruxism, although they have been widely used in many studies on the diagnosis, prevalence, and treatment of bruxism [20,55,56,57,58,60,61,62,63,64].

According to our results, both manual therapy and home exercise treatment were found to be significantly effective in reducing bruxism, TMD symptoms, perceived stress, pain, and trigger point number while increasing sleep quality and quality of life. However, although the improvements in the manual therapy group were relatively higher than the exercise group, it was remarkable that both methods had statistically similar effects in terms of quality of life, pain, and perceived stress. In terms of bruxism score, FAI, sleep quality, and number of trigger points, there was a statistically greater improvement in the manual therapy group. This result suggests that manual therapy may be needed more in more severe bruxism cases. It will be useful to support these results with electromyography and ultrasound studies, which we plan to conduct in the future. In addition, this study aimed to examine the short-term effects of manual therapy and home exercises in bruxism patients. These results are the outcomes of 8 weeks of active physical therapy, and long-term studies on the stability of these changes in the future will contribute to our results.

As it is known, the incidence of bruxism decreases with age, while the incidence of TMD increases [4,7,8,99]. For this reason, our study was conducted on a group of young adult patients ages 18–25 years. One of the objectives of the study was to determine the effect of manual therapy and exercise therapy on stress and quality of life in bruxist individuals. For this reason, we aimed to ensure similarity and homogeneity between the randomized treatment groups by including participants who were studying at the same university, were in the same age group, lived in similar social environments, and had similar stress levels. Thus, we tried to reduce the diversity that may affect subjective data such as stress and quality of life, which are closely related to many factors. For this reason, different results can be expected in studies conducted in different age groups and different populations.

Bruxism is often difficult to diagnose directly by patients because of how it occurs [100]. International consensus on the assessment of bruxism has proposed the use of a grading system that defines a diagnosis of sleep or awake bruxism as “possible” based on self-report, “probable” based on clinical examination, or “definite” based on a polysomnographic record [3]. Non-instrumental approaches for assessing bruxism include self-report (questionnaires, oral history) and clinical inspection. In this study, bruxism was diagnosed using both a questionnaire and clinical examination to determine “probable bruxism”. Although polysomnography is considered to be the gold standard for the definitive diagnosis of sleep bruxism, its use in clinical practice has not been possible due to its high cost and the need for specialized equipment [101]. A study which aimed to determine the agreement of patient self-reports and clinical signs of sleep bruxism with electromyographic/electrocardiographic data indicated that self-reported questionnaires and clinical signs have moderate sensitivity, specificity, and accuracy for diagnosing bruxism comparing with an ambulatory device for current sleep bruxism [102].

Existing data show that there is a positive relationship between bruxism and TMD and that the presence of bruxism may also have psychosocial effects such as pain, stress, and sleep disorders [103]. Achieving significant improvements thanks to the manual therapy and home exercises applied in our study is important in terms of preventing the occurrence of physical, emotional, behavioral, and psychosocial disorders that may be seen in the future, as well as early diagnosis and awareness of bruxism and possible TMD problems.

The fact that manual therapy and home exercise therapy are non-invasive and do not involve any medication makes them a good alternative for the treatment of bruxism for children and pregnant or breastfeeding women and provides easy use without any side effects. In this context, the home exercise application, which is more economical, easily accessible, and applicable and shows similar effects to manual therapy, can be given a wider place in the treatment of bruxism. In addition to dentist/psychologist applications, such as occlusal splint therapy and cognitive therapy, home exercises can be easily included in the treatment plan, and more multidisciplinary clinical studies can be conducted on them.

### Limitations

As in all studies conducted on bruxism and TMD, the number of women in both groups was higher than men in our study, but it is thought that the statistical similarity of the groups in terms of gender ensures the homogeneity of the groups.

During the formation of the study group, individuals with bruxism were selected according to a clinical evaluation by the specialist dentist and physiotherapist as well as the bruxism questionnaire. The polysomnography method, which is the gold standard in the diagnosis of bruxism, could not be used since it is uneconomical and inaccessible. However, the participants were evaluated twice by both the specialist physiotherapist and the specialist dentist to confirm the diagnosis of bruxism.

The study consisted only of treatment groups, and a control group without treatment was not included in the study. Although this makes it difficult to make comparative analyses, it is considered unethical to keep a patient with bruxism, which is painful and has various negative psychosocial effects, waiting without providing any treatment.

Another limitation of our study is the moderate sample size, relatively short time frame, and lack of long-term follow-up. However, the promising results obtained in this study provide a strong rationale for expanding the sample size and duration of future studies. Replicating this study in larger samples could validate the beneficial results obtained with manual therapy and home exercise interventions and explore the potential for use in various populations.

In future studies, it would be useful to examine longer-term effects in different age groups and individuals with different demographic characteristics using a larger sample size.

## 5. Conclusions

- It can be concluded that manual therapy and home exercise treatment for bruxism reduce the pain and perceived stress and increase the quality of life and sleep.
- Home exercise is as effective as manual therapy in terms of improvement in pain, perceived stress, and quality of life.
- Manual therapy is more effective than home exercise in reducing bruxism and TMD symptoms and improving sleep quality.
- Manual therapy and home exercise can be applied as alternative treatment methods in the treatment of bruxism.

## Figures and Tables

**Table 1:** Bruxism questionnaire.

Bruxism Questionnaire	YES	NO
Does anyone hear you grinding your teeth at night?		
Do you feel fatigue or pain in your jaw when you wake up in the morning?		
Do you feel pain in your teeth and gums when you wake up in the morning?		
Do you have a headache when you wake up in the morning?		
Do you notice that you grind your teeth during the day?		
Do you notice that you clench your teeth during the day?		

**Table 2:** Fonseca Anamnestic Index.

Fonseca Anamnestic Index	Yes	Sometimes	No
Do you have difficulty opening your mouth widely?			
Do you have difficulty moving your lower jaw left or right?			
Do you feel muscle fatigue/pain when chewing?			
Do you often have headaches?			
Do you have neck pain or neck stiffness?			
Do you have pain in your ear or jaw joint?			
Do you hear any clicking sounds from the jaw joint when chewing or opening your mouth?			
Do you have a habit of clenching or grinding your teeth?			
Do you feel that your teeth do not come together well?			
Do you think you are a nervous (irritable) person?			

Classification of TMD severity: 0–15 points = No TMD; 20–40 points = Mild TMD; 45–65 points = Moderate TMD; 70–100 points = Severe TMD.

**Table 3:** Sociodemographic characteristics of each group and result of Pearson’s Chi-squared test.

Sociodemographic Characteristics	MTG (*n* = 15)		HEG (*n* = 15)		Analysis
	*n*	%	*n*	%	
Gender					
Female	11	73.3	10	66.7	χ^2^ = 0.159
Male	4	26.7	5	33.3	*p* = 0.690
Age					
18–20	6	40	7	46.7	χ^2^ = 4.667 *p* = 0.587
21–22	7	46.7	4	26.6	
23 and up	2	13.3	4	26.6	
Side used for chewing					
Right	6	40	5	33.3	χ^2^ = 1.691 *p* = 0.429
Left	3	20	1	6.7	
Both sides	6	40	9	60	
Do you suffer from headaches?					
Yes	13	86.7	13	86.7	
No	2	13.3	2	13.3	
Do you feel stiffness in your neck?					
Yes	10	66.7	12	80	χ^2^ = 0.682 *p* = 0.409
No	5	33.3	3	20	
Do you have any parafunctional habits?					
Yes	15	100	15	100	χ^2^ = 1.033 *p* = 0.309
No	0	0	0	0	

MTG: manual therapy group, HEG: home exercise group, *n*: number of participants, χ^2^: Pearson’s Chi-squared test, *p* < 0.05.

**Table 4:** Pre-treatment (T1) and post-treatment (T2) comparative findings regarding sleep quality, quality of life, and perceived stress level in manual therapy and home exercise groups.

		MTG (*n*: 15) X ± SD	HEG (*n*: 15) X ± SD	Difference Between Groups
Pittsburgh Sleep Quality Index (PSQI)	T1	11.40 ± 3.37	7.86 ± 2.99	t = 0.305 ^†^ *p* = 0.763 ^†^
	T2	6.26 ± 2.54	6.00 ± 2.23	t = 3.031 ^†^ *p* = 0.005 ^†^
	Difference within group (T1–T2)	t = 10.148 *p* < 0.001	t = 2.736 *p* = 0.016	
Quality of Life/ Short Form-36 (SF-36)	T1	45.00 ± 11.33	51.33 ± 20.39	t = −1.051 ^†^ *p* = 0.302 ^†^
	T2	67.33 ± 21.11	58.00 ± 16.88	t = 1.337 ^†^ *p* = 0.192 ^†^
	Difference within group (T1–T2)	t = −4.580 *p* < 0.001	t = −2.870 *p* = 0.012	
Perceived Stress	T1	19.67 ± 2.16	18.46 ± 2.87	t = 1.292 ^†^ *p* = 0.207 ^†^
	T2	15.27 ± 5.75	15.53 ± 3.04	t = −0.159 ^†^ *p* = 0.875 ^†^
	Difference within group (T1–T2)	t = 2.710 *p* = 0.017	t = 3.556 *p* = 0.003	

MTG: manual therapy group, HEG: home exercise group, *n*: number of participants, X ± SD: mean and standard deviation, T1: before treatment, T2: after treatment, Difference within group (T1–T2): paired samples *t* test, Difference between groups: independent *t* test (^†^), *p* < 0.05.

**Table 5:** Pre-treatment (T1) and post-treatment (T2) comparative findings regarding Fonseca Anamnestic Index, bruxism questionnaire, number of trigger points, and pain level in manual therapy and home exercise groups.

		MTG (*n*: 15) X ± SD	HEG (*n*: 15) X ± SD	Difference Between Groups
Fonseca Anamnestic Index (FAI)	T1	67.67 ± 11.32	71.33 ± 13.02	t = −0.823 ^†^ *p* = 0.417 ^†^
	T2	34.33 ± 13.61	44.33 ± 12.80	t = −2.073 ^†^ *p* = 0.047 ^†^
	Difference within group (T1–T2)	t = 7.468 *p* < 0.001	t = 10.894 *p* < 0.001	
Bruxism Questionnaire	T1	4.66 ± 1.17	3.60 ± 1.35	t = 2.306 ^†^ *p* = 0.029 ^†^
	T2	1.00 ± 1.00	1.73 ± 1.33	t = −1.703 ^†^ *p* = 0.100 ^†^
	Difference within group (T1–T2)	t = 7.892 *p* < 0.001	t = 7.897 *p* < 0.001	
Number of Trigger Points	T1	10.26 ± 2.18	10.26 ± 1.62	t = 0.000 ^†^ *p* = 1.000 ^†^
	T2	5.73 ± 2.05	7.80 ± 2.36	t = −2.556 ^†^ *p* = 0.016 ^†^
	Difference within group (T1–T2)	t = 11.311 *p* < 0.001	t = 4.281 *p* < 0.001	
Rest Pain	T1	4.64 ± 2.61	3.50 ± 2.22	t = 1.294 ^†^ *p* = 0.206 ^†^
	T2	1.26 ± 1.38	1.96 ± 2.25	t = −1.024 ^†^ *p* = 0.315 ^†^
	Difference within group (T1–T2)	t = 4.649 *p* < 0.001	t = 1.994 *p* = 0.066	
Activity Pain	T1	5.08 ± 2.62	4.53 ± 2.50	t = 0.587 ^†^ *p* = 0.562 ^†^
	T2	2.06 ± 2.49	2.96 ± 2.53	t = −0.981 ^†^ *p* = 0.335 ^†^
	Difference within group (T1–T2)	t = 4.490 *p* < 0.001	t = 3.022 *p* = 0.009	
Night Pain	T1	4.50 ± 3.65	4.10 ± 2.94	t = 0.330 ^†^ *p* = 0.744 ^†^
	T2	1.40 ± 2.09	2.70 ± 2.39	t = −1.581 ^†^ *p* = 0.125 ^†^
	Difference within group (T1–T2)	t = 3.470 *p* = 0.004	t = 3.004 *p* = 0.009	

MTG: manual therapy group, HEG: home exercise group, *n:* number of participants, X ± SD: mean and standard deviation, T1: before treatment, T2: after treatment, Difference within group (T1–T2): paired samples *t* test, Difference between groups: independent *t* test (^†^), *p* < 0.05.

## Data Availability

The data supporting this research are available upon request from the corresponding authors for data protection reasons.

## Supplementary Materials

The following supporting information can be downloaded at: https://www.mdpi.com/article/10.3390/medicina60122007/s1, Supplementary S1: Power analysis for sample size calculation; Supplementary S2: Shapiro-Wilk test of normality.
