# Supplementary material for: Effects of Manual Therapy and Home Exercise Treatment on Pain, Stress, Sleep, and Life Quality in Patients with Bruxism: A Randomized Clinical Trial
Source: Medicina (Kaunas). 2024 Dec 4;60(12):2007. doi: 10.3390/medicina60122007 (PMC11727747; doi:10.3390/medicina60122007)
Supplement: Supplementary file 1 [file medicina-60-02007-s001.zip › medicina-3276839-supplementary.pdf]

Supplementary S1: Power analysis for sample size calculation.

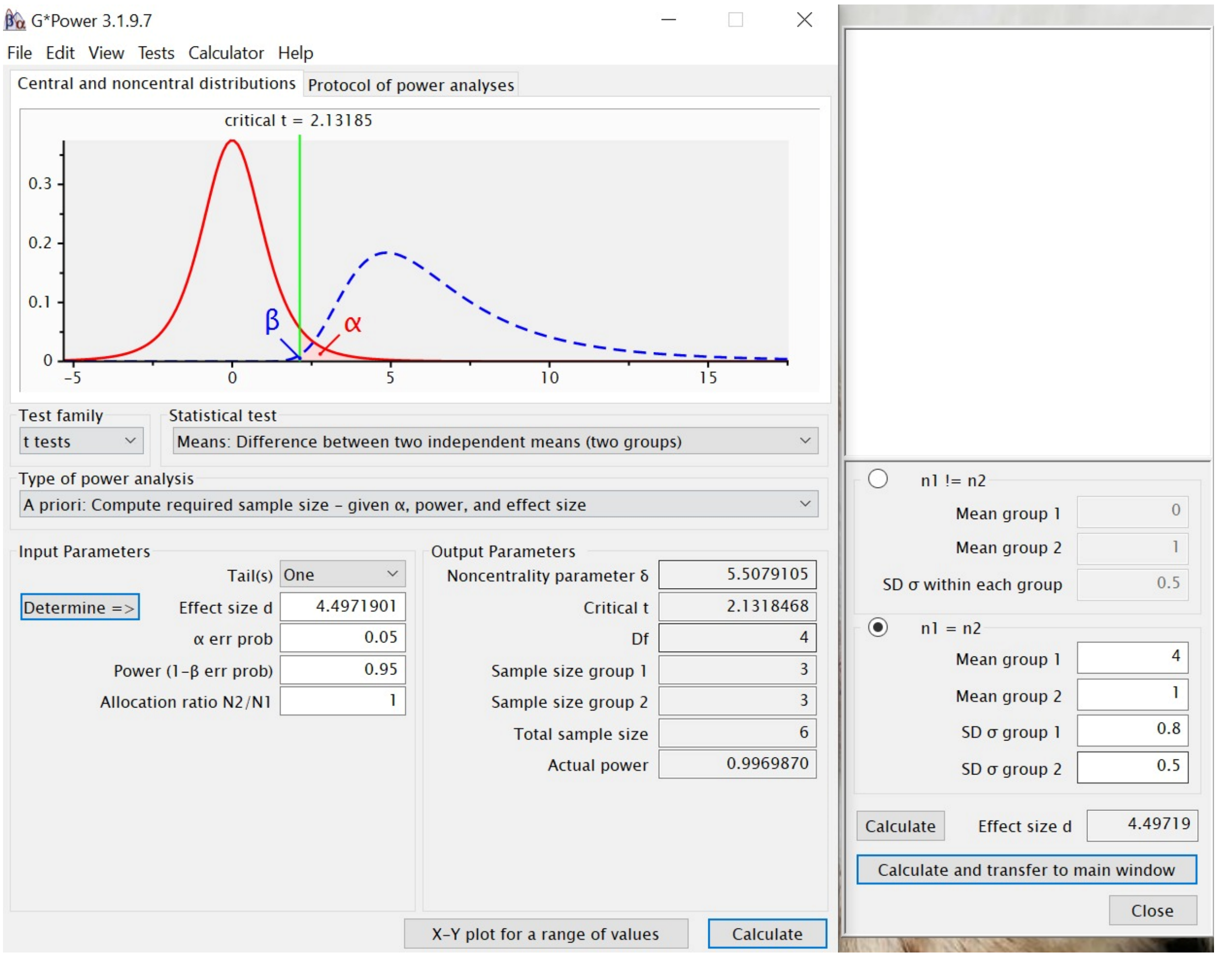

Supplementary S2: Table of Shapiro-Wilk test for normality.

	Test of Normality/Shapiro-Wilk	Statistic	df	Sig.
Home Exercise Group (HEG)	Fonseca Anamnestic Index	0,952	15	0,564
	Total Trigger Point	0,910	15	0,136
	Perceived Stress Scale	0,954	15	0,592
	Pittsburgh Sleep Quality Index (PSQI):	0,908	15	0,124
	Quality of Life Scale / Short Form-36 (SF-36)	0,961	15	0,702
	Visual Analog Scale - Rest	0,880	15	0,057
	Visual Analog Scale - Activity	0,931	15	0,278
	Visual Analog Scale - Night	0,883	15	0,052
Manual Therapy Group (MTG)	Fonseca Anamnestic Index	0,888	15	0,063
	Total Trigger Point	0,930	15	0,273
	Perceived Stress Scale	0,923	15	0,213
	Pittsburgh Sleep Quality Index (PSQI):	0,982	15	0,983
	Quality of Life Scale / Short Form-36 (SF-36)	0,969	15	0,841
	Visual Analog Scale - Rest	0,970	15	0,851
	Visual Analog Scale - Activity	0,901	15	0,099
	Visual Analog Scale - Night	0,893	15	0,074
